# Cisplatin loaded multiwalled carbon nanotubes reverse drug resistance in NSCLC by inhibiting EMT

**DOI:** 10.1186/s12935-021-01771-9

**Published:** 2021-01-25

**Authors:** Yuxin Qi, Wenping Yang, Shuang Liu, Fanjie Han, Haibin Wang, Yonghong Zhao, Yufa Zhou, Daijun Zhou

**Affiliations:** 1 Department of Respiratory Medicine, Jinan People’s Hospital Affiliated to Shandong First Medical University, Jinan, 271199 China; 2 Department of Oncology, General Hospital of Western Theater Command of PLA, Chengdu, 610083 China

**Keywords:** Epithelial-mesenchymal transformation, Non-small-cell lung cancer, 5-year survival rate, Chemotherapy

## Abstract

**Background:**

Lung cancer is one of the important health threats worldwide, of which 5-year survival rate is less than 15%. Non-small-cell lung cancer (NSCLC) accounts for about 80% of all lung cancer with high metastasis and mortality.

**Methods:**

Cisplatin loaded multiwalled carbon nanotubes (Pt-MWNTS) were synthesized and used to evaluate the anticancer effect in our study. The NSCLC cell lines A549 (cisplatin sensitive) and A549/DDP (cisplatin resistant) were used in our in vitro assays. MTT was used to determine Cancer cells viability and invasion were measured by MTT assay and Transwell assay, respectively. Apoptosis and epithelial-mesenchymal transition related marker proteins were measured by western blot. The in vivo anti-cancer effect of Pt-MWNTs were performed in male BALB/c nude mice (4-week old).

**Results:**

Pt-MWNTS were synthesized and characterized by X-ray diffraction, Raman, FT-IR spectroscopy and scan electron microscopy. No significant cytotoxicity of MWNTS was detected in both A549/DDP and A549 cell lines. However, Pt-MWNTS showed a stronger inhibition effect on cell growth than free cisplatin, especially on A549/DDP. We found Pt-MWNTS showed higher intracellular accumulation of cisplatin in A549/DDP cells than free cisplatin and resulted in enhanced the percent of apoptotic cells. Western blot showed that application of Pt-MWNTS can significantly upregulate the expression level of Bax, Bim, Bid, Caspase-3 and Caspase-9 while downregulate the expression level of Bcl-2, compared with free cisplatin. Moreover, the expression level of mesenchymal markers like Vimentin and N-cadherin was more efficiently reduced by Pt-MWNTS treatment in A549/DDP cells than free cisplatin. In vivo study in nude mice proved that Pt-MWNTS more effectively inhibited tumorigenesis compared with cisplatin, although both of them had no significant effect on body weight.

**Conclusion:**

Pt-MWNT reverses the drug resistance in the A549/DDP cell line, underlying its possibility of treating NSCLC with cisplatin resistance.

## Background

Lung cancer is one of the leading causes of deaths all over the world [1]. Non-small cell lung cancer (NSCLC) is the most common histological type of lung cancer [2]. It has high morbidity due to its early recurrence and widespread metastatic potential [3], with the 5-year overall survival rate less than 5% [4]. A number of factors may increase the risk of lung cancer, such as air pollution and smoking [5].

In the last few years, many progresses were made in the treatment of lung cancer [6], among these platinum-based chemotherapy is most commonly used, especially for patients in advanced stages [7]. Cisplatin is one of the chemotherapeutic drugs widely used in clinic. It has shown anticancer activity in a variety of tumors including cancers of the ovaries, lung, and solid tumors of the head and neck [8–10]. However, the sensitivity of tumor cells to cisplatin will be significantly reduced after long-term use, resulting in acquired drug resistance [11]. The underlying mechanisms of drug resistance were complicated. Platinum-uptake rely on multiple transporters. Dysregulation of primary transporters were responsible for cisplatin resistance by influencing platinum cell accumulation [12–15]. Besides, increased detoxification system was also a reason for cisplatin resistance. Elevated expression of glutathione reductase is often seen in the resistant-cells [16]. Chelating with metallothionein (MT) proteins can also inactivate the anti-tumor activity of platinum [17]. Moreover, platinum can form platinum–DNA adducts and the increasement of DNA repair process is the most prominent feature of platinum-resistance cells which cause cell resistance [18, 19]. Decreased apoptosis and increased autophagy also responsible for platinum-resistant, since platinum-resistant tumor cells usually have a lower level of apoptosis induction and increased autophagy [20–23].

In addition to the above, epithelial-mesenchymal transition (EMT) is a process underlying the malignant progression of carcinoma, and is also one of the important mechanisms of cisplatin resistance in tumor cells [24]. Recently, study shows that tumor cells acquire characteristics of invasiveness and metastasis through EMT and lead to increased resistance to antitumor drugs [25–27]. Yang et al. found that Jagged1 can cooperate with the JAK/STAT3 pathway to promote EMT and further facilitate the invasion and migration of platinum-resistant ovarian cancer cells [28]. Besides, a previous study reported that the switch expression of CD44 variant in oral cancer resulted in tumor cell cisplatin-resistance by inducing EMT progress [29]. These results indicated that EMT is of great importance during tumor cells platinum resistance process and highlighted the potential of targeting EMT in clinical application of platinum. In this study, we used carbon nanotubes to load cisplatin and investigated its potential to reverse platinum resistance in NSCLC by inhibiting EMT progress.

## Materials and methods

### Cell culture

The NSCLC cell lines A549, A549/DDP were purchased from Cell Bank of Chinese Academy of Sciences (Shanghai, China). A549 and A549/DDP Cells were cultured in PRIM 1640 (GIBCO, NY, USA) containing 10% fetal bovine serum (Biological Industries) and 1% penicillin/streptomycin at 37℃ under 5% CO2.

### Preparation and characterization of Pt-MWNTS

MWNTS were synthesized according to a previously report [30]. MWNTS were characterized by X-ray diffraction, FT-Raman and FT-IR spectrometry. To prepare Pt-MWCNTs, cisplatin was mixed with MWNTS dispersed in PBS and stirred overnight at room temperature in dark conditions. Unloaded cisplatin was removed by thoroughly dialyzing the reaction mixture against PBS. Encapsulation efficiency and drug-loading capacity of cisplatin onto MWNTS were quantified at 254 nm by UV–vis spectroscopy based on a standard curve of cisplatin.

### MTT assay

3-(4,5-dimethylthiazol-2yl)-2,5-diphenyltetrazolium bromide (MTT) was used to determine cells viability. A549 and A549/DDP cells were seeded in 96-well plates. Then cells were treated with different concentration of Pt and Pt-MWNTS at 0.01, 0.01, 0.1, 1, 10, 100 μM for 48 h, followed by incubation with MTT for 4 h. 100 μl dimethyl sulfoxide was added to each well to dissolve the formazan crystals. Finally, absorbance at 490 nm were measured in a microplate reader.

### Colony formation assay

About 500 A549 or A549/DDP cells were seeded in 10-cm dishes containing complete medium and drugs as indicated. The plates were gently put in CO2 incubator. After 7 days, the colonies were stained with 0.1% crystal violet and counted.

### Transwell assays

For transwell assay, the upper chamber was added with DMEM culture solution (200 μL; containing 3 × 10^4^ cells), while the lower chamber was added with DMEM (500 μL; containing 20% FBS). After incubation for 24 h at 37℃ under 5% CO2, the cells that did not penetrate the membrane surface in the upper chamber were wiped off. And the cells in the lower chamber were washed with PBS for 3 times, and fixed with paraformaldehyde for 10 min, and finally stained with 0.1% crystal violet. The number of cells were counted under a light microscope (Olympus, Tokyo, Japan).

### HPLC

To analyze the intracellular cisplatin, cells were harvested and washed by serum-free medium for 3 times. The supernatant was discarded and resuspended in 0.3 ml of distilled water. Cells were lyzed by repeated freeze–thaw, and the supernatant was centrifuged at 10,000 rpm/min for 30 min. The detection was performed by C18 chromatographic column use cisplatin as a standard. Mobile phase was methanol: water (prepared according to the volume ratio of 2:1). The flow rate was 1.0 ml/min. The ultraviolet detection wavelength was 254 nm, and the detection sensitivity was 0.01AuFS.

### Western blot

The cells were collected by a mixture of RIPA buffer and protease inhibitor (100:1). To get total protein, cells were broken up by Selecta Sonopuls and centrifuged after ultrasonication. Protein concentration was determined by BCA method. Protein samples were separated by polyacrylamide gel and then transferred onto NC membrane (Millipore). The membrane was blocked in 5% nonfat milk at RT for 1 h, and then probed with primary antibodies. Primary antibodies against bax (1: 1000, GeneTex), bcl2 (1: 1000, GeneTex), caspase-3 (1: 1000, GeneTex), and casapase (1: 1000, GeneTex) were incubated overnight at 4 °C. Secondary antibodies (1: 10,000, abcam) was incubated for 50 min. Bands were visualized using Western Bright ECL (Advansta) and captured with ImageQuant Chemiluminescent Imaging System, LAS 500 (GE Healthcare Bio-sciences AB). The relative band intensity was quantified with imageJ2 software and normalized to β-actin.

### Nude mice

Animal study was approved by the Ethics Committee of Jinan People’s Hospital Affiliated to Shandong First Medical University. Male BALB/c nude mice (4-week old) were maintained in pathogen-free conditions. Nude mice were injected subcutaneously with 5 × 10^5^ A549/DDP cells in 100 μl PBS for tumor formation. Normal saline served as the control. The next day, Pt and Pt-MWNTS were administrated by intravenous injection. Tumors were removed for assessments after 18 days.

### Statistical analysis

The statistical analyses were performed using a two-tailed Student's paired t-test and one-way ANOVA. P values of less than 0.05 were considered significant. Experiments were repeated more than 3 times.

## Results

### Characterization of Pt-MWNTS

**Fig. 1:**
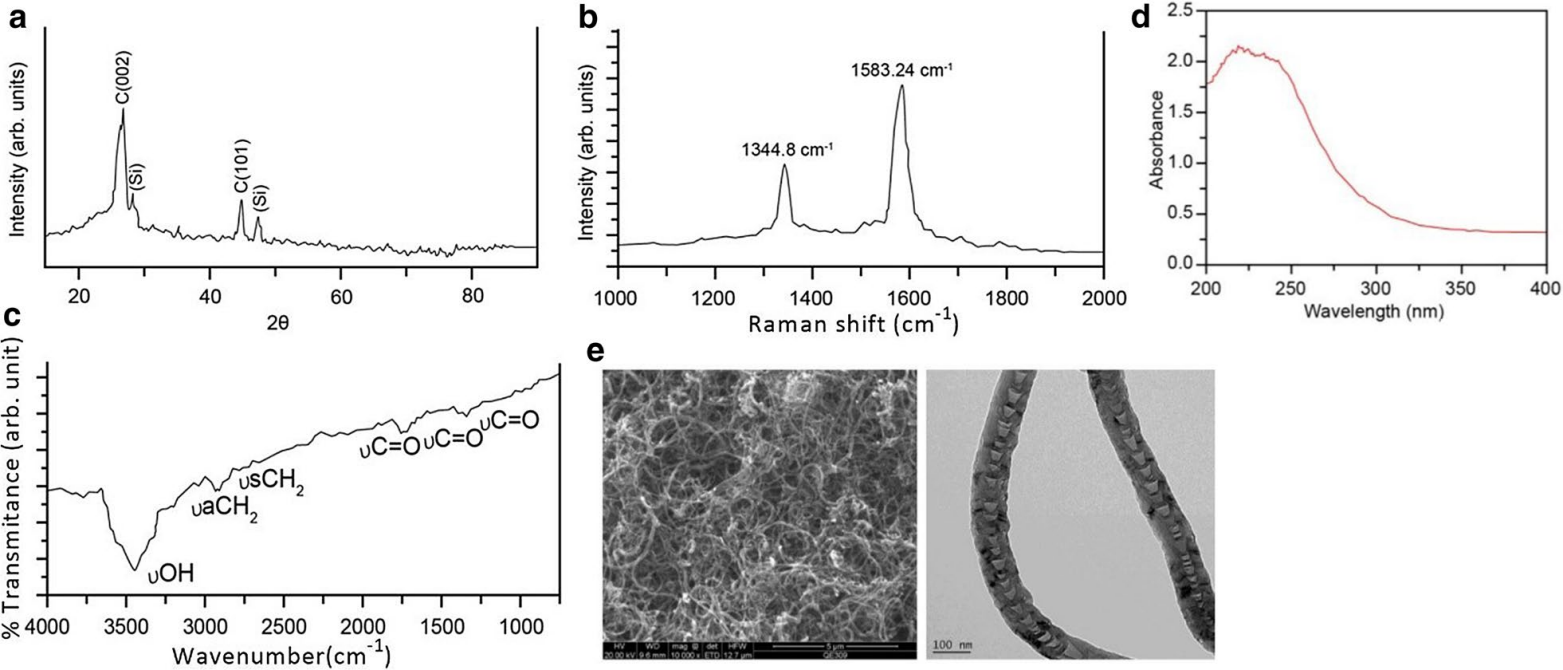
Synthesis and characterization of Pt-MWNTS. **a** X-ray diffraction of purified MWNTS; **b** FT-Raman spectroscopy of purified MWNTS; **c** FT-IR spectroscopy of purified MWNTS; **d** UV − vis spectra of cisplatin loaded MWNTS showing the strong absorbance of cisplatin around 250 nm; **e** High resolution scanning electron microscopy of cisplatin loaded MWNTS

Carbon nanotubes (CNTs) are a kind of new carbon-based nanomaterials (NMs) that could be used in lots of areas from electronics to biotechnology [31]. Carbon-based materials including multiwall carbon nanotubes (MWCNTs) recently have attracted significant attention in a series of reports concerning their potential use in cancer treatment [32, 33]. MWNTS were synthesized according to a previously reported method [30] and loaded with cisplatin to generate Pt-MWNTS. Figure 1a shows the outlook of Pt-MWNTS. Figure 1b shows the TEM of Pt-MWNTS. The absorption spectrum of Pt-MWNTS shows a strong peak between 200 and 300 nm (Fig. 1c–e).

### Anticancer effect of Pt-MWNTS on cisplatin-resistance cell lines

**Fig. 2:**
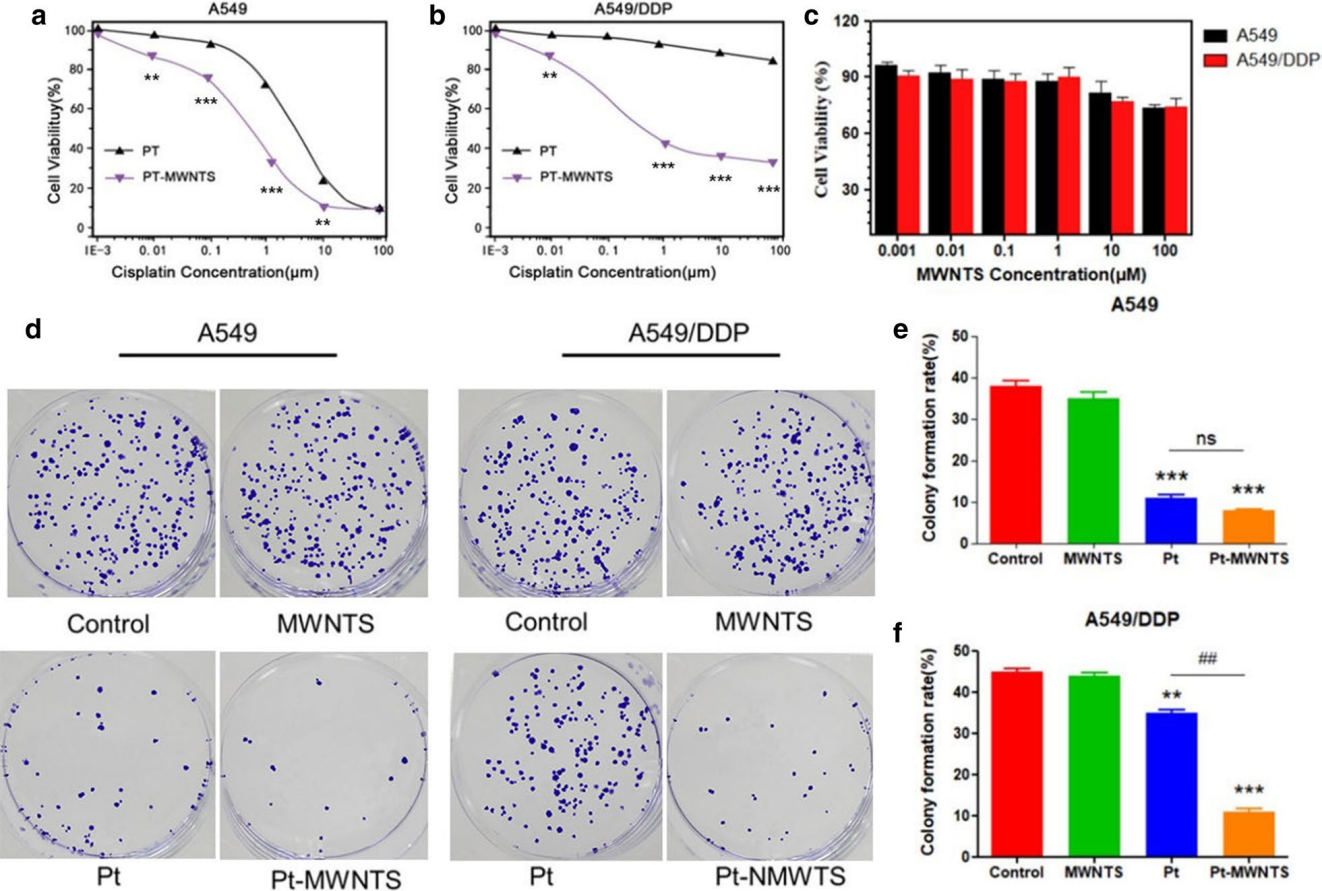
Pt-MWNTS reverse drug resistance and affect colony formation of A549/DDP. MTT assay of NSCLC cell lines **a** A549 and **b** A549/DDP treated by Pt and Pt-MWNTS at different cisplatin concentration for 48 h (n = 5). **c** Using different concentration of MWNTS to treat A549 and A549/DDP cells for 48 h (n = 5 per group). **d**–**f** Colony formation assay of A549 and A549/DDP cells. Cells were seed in 10-cm dish at a density of 500 cells/dish. Then cells were treated with PBS (control), MWNTS, cisplatin (Pt) and Pt-MWNTS at a concentration of 50 μM. After 7 days, we stained the dishes with crystal violet to count the number of colonies. The colony formation rate was calculated by divide the total colony number in each well by 500. (n = 3, **P < 0.01, ***P < 0.001 vs Control; ^##^*P* < 0.01 vs Pt.)

To evaluate the anticancer efficacy of Pt-MWNTS, it was incubated with human non-small cell lung cancer cell line (A549) and human cisplatin-resistant lung adenocarcinoma cell line (A549/DDP) at different doses for 48 h. MTT assay indicated that A549 cells were highly susceptible to cisplatin and Pt-MWNTS, while A549/DDP cells displayed resistance to cisplatin, but was sensitive to Pt-MWNTS, suggesting that Pt-MWNTS can significantly reverse cisplatin resistance (Fig. 2a, b). To see if the observed anticancer effect of Pt-MWNTS was due to the intrinsic cytotoxicity of MWNTS, the cytotoxicity of MWNTS at the same concentration was also evaluated (Fig. 2c). No significant cytotoxicity was detected in both A549/DDP and A549 cell lines, indicating that the enhanced anti-tumor proliferation activity of Pt-MWNTS was due to the loaded cisplatin. To further test the inhibition effect on cell growth, colony formation assays were performed using cisplatin and Pt-MWNTS. As shown in Fig. 2d, both drugs had similar inhibition effects on A549 cells. Importantly, a stronger inhibition effect of Pt-MWNTS on cell growth was observed in A549/DDP cells. Overall, these data suggested that Pt-MWNTS could inhibit the growth of cisplatin-resistant cancer cells (Fig. 2).

### Cellular uptake of Pt-MWNTS

**Fig. 3:**
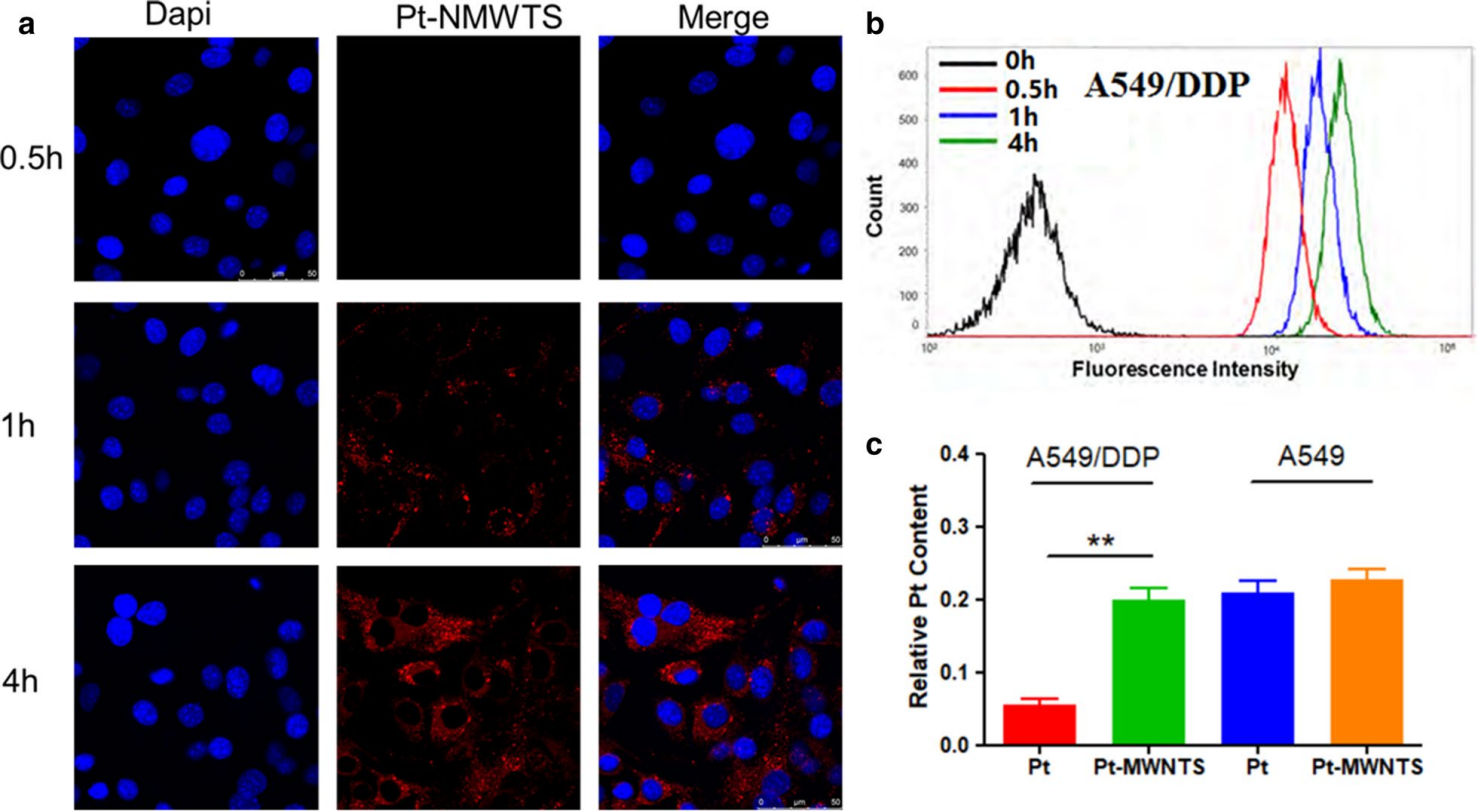
Endocytosis and cellular concentration of Pt-MWNTS in non-small cell lung cancer cells. **a** Representative images of confocal microscopy showed the intracellular localization of Pt-NMWTS. (Pt-NMWTS is stained in red and the nuclei is in blue, scale bar = 50 μm). **b** The endocytosis of Pt-NMWTS was observed by flow cytometry at a time point of 0, 0.5, 1, and 4 h. **c** Relative intracellular uptake based on Pt was also quantitatively measured after 4 h treatment of Pt and Pt-NMWTS at 50 μM A549 and A549 DDP cells respectively. (n = 3, **P < 0.01 vs Pt.)

In order to track the cellular internalization and intracellular distribution of Pt-MWNTS in A549/DDP, the cells were treated with Rhodamine B labeled Pt-MWNTS and observed the cells by confocal laser scanning microscopy (Fig. 3a). Moreover, the uptake of Pt-MWNTS was also studied via fluorescently labeled Pt-MWNTS by flow cytometry (Fig. 3b). The results showed that cellular uptake of Pt-MWNTS was increased from 1 to 4 h in a time-dependent manner. Pt-MWNTS were mainly distributed in the cytoplasm. Then, we compared the intracellular accumulation of cisplatin and Pt-MWNTS by HPLC. As shown in Fig. 3c, Pt-MWNTS showed higher intracellular accumulation of cisplatin in A549/DDP cells. Overall, these data suggested that Pt-MWNTS facilitate the uptake of the loaded cisplatin into the cytoplasm.

### Underlying mechanism of Pt-MWNTS on cellular toxicity

**Fig. 4:**
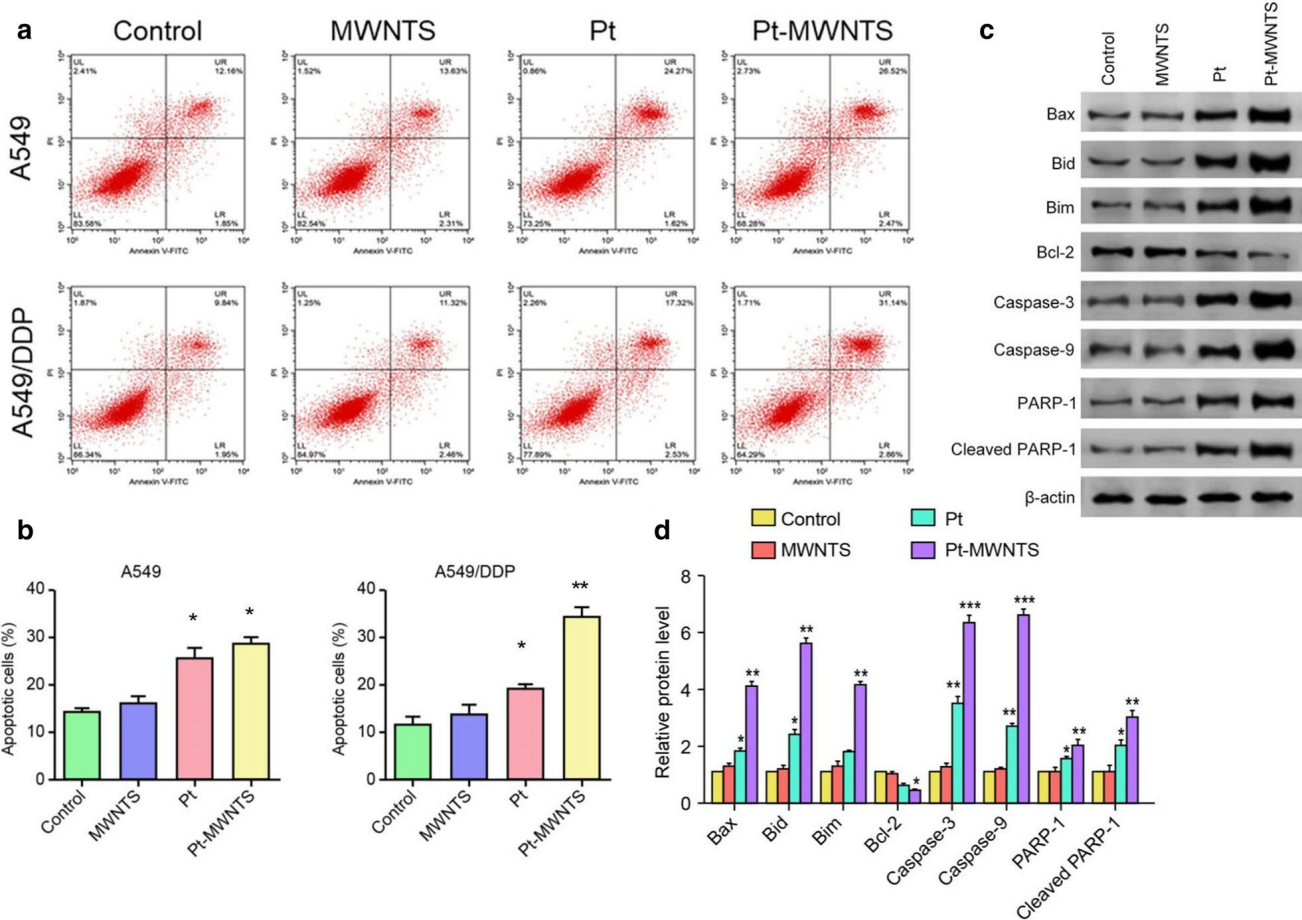
Pt-MWNTS induced cell apoptosis of A549 & A549/DDP. **a** Representative flow cytometry data for the detection of apoptosis in A549 and A549/DDP cells treated with PBS, MWNTS, cisplatin (Pt) and Pt-MWNTS at 30 μg/ml. **b** Quantification of the percent of apoptotic cells in each treatment group. **c** Representative western blot showing the protein levels of apoptosis markers Bax, Bid, Bim, Bcl-2, Caspase-3, Caspase-9 and PAPR-1. β-actin served as loading control. A549/DDP cells were treated under different drug concentration for 48 h. **d** Quantification of the relative amount of each protein detected by western blot. Data represented mean $\pm$ SD of three independent experments. (n = 3, *P < 0.05*,* **P < 0.01, ***P < 0.001 vs Control.)

To study the mechanism of Pt-MWNTS, Annexin-V/PI staining of the A549 and A549/DDP cells was performed for the detection of apoptosis. Cells treated with Pt-MWNTS showed higher apoptosis rate, containing both early and late stage apoptosis (Fig. 4a). On the contrary, the apoptosis rate was lower in the cisplatin treatment group, especially in A549/DDP cells (Fig. 4b). Western blot results also showed that application of cisplatin and Pt-MWNTS can significantly upregulate the expression level of Bax, Bim, Bid, Caspase-3, Caspase-9 and PAPR-1 while the expression level of Bcl-2 was downregulated (Fig. 4c, d and Additional file 1: Fig. S1). These data revealed that Pt-MWNTS showed higher pro-apoptotic effect than free cisplatin.

**Fig. 5:**
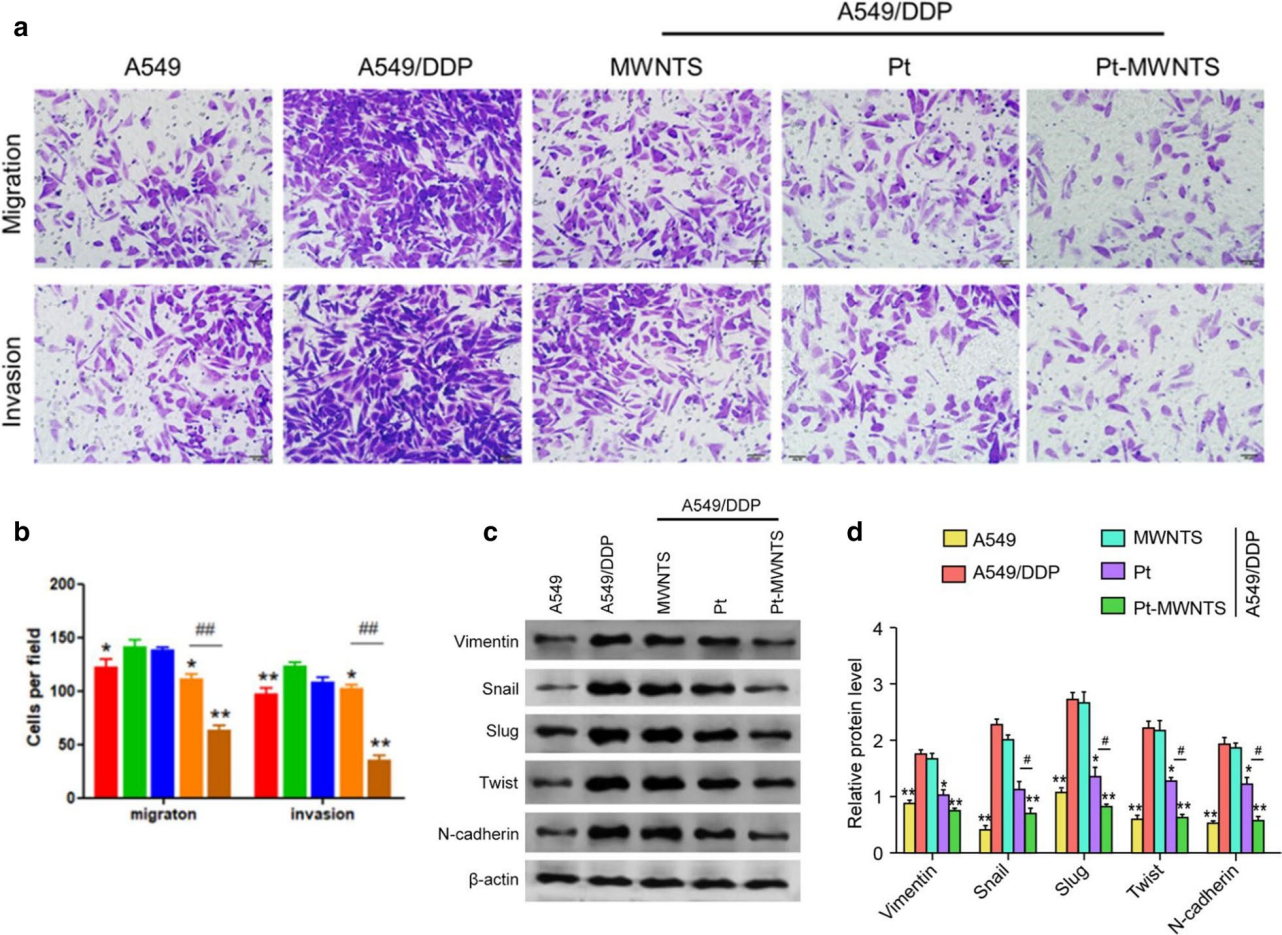
Pt-MWNTS effects on NSCLC cells migration, invasion and EMT progress. **a** Representative images of NSCLC cells migration and invasion by transwell assay. **b** Quantification of the cell migration and invasion. **c** The expression levels of EMT related markers Vimentin, Slug, Snail, Twist and N-cadherin were detected by western blot. Representative images were shown. A549/DDP cells were treated at different drug formulations for 48 h. **d** Quantification of the relative protein levels based on western blot data. (n = 3, *P < 0.05*,* **P < 0.01 vs A549/DDP; ^#^*P* < 0.05, ^##^*P* < 0.01 vs Pt)

Moreover, transwell assay was performed to test the effect on cell invasion. We found that cisplatin-resistant lung adenocarcinoma cell line (A549/DDP) show enhanced migration and invasion ability than A549, and Pt-MWNTS could significantly inhibit its migration and invasion ability (Fig. 5a, b). EMT is one of the important mechanisms of cisplatin resistance in tumor cells [27], thus we hypothesized that Pt-MWNTS inhibit cisplatin-resistant cells growth by regulating EMT progress. We first compared the EMT related markers expression level between A549 cell lines and A549/DDP cell lines. As shown in Fig. 5c, d, cisplatin-resistant cell line A549/DDP showed higher expression level of mesenchymal markers like Vimentin and N-cadherin than that of A549 cells. Meanwhile, the protein expression levels of EMT-induced transcription factors Snail, Slug and Twist1 were also significantly increased. After the addition of cisplatin or Pt-MWNTS to A549/DDP cells, the expression of EMT interstitial markers and transcription factors was inhibited at different degrees and the effect of Pt-MWNTS was better than cisplatin, indicating that Pt-MWNTS could inhibit the occurrence of EMT by regulating the interstitial markers and transcription factors in the process of EMT.

### Application of Pt-MWNTS inhibits tumorigenesis in vivo

**Fig. 6:**
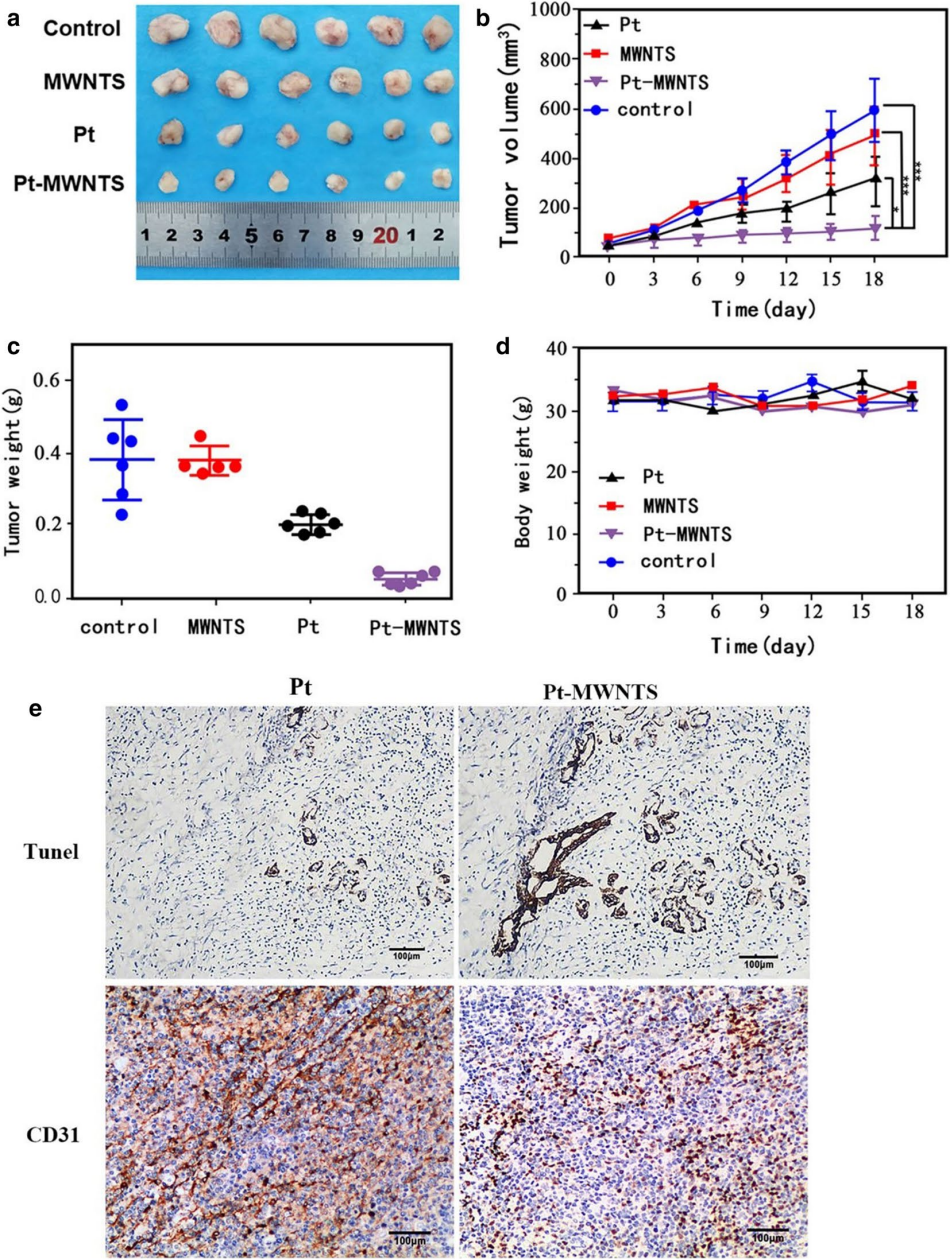
Pt-MWNTS inhibit tumorigenesis in vivo. **a** Representative photographs of tumorigenesis via subcutaneous injection of A549/DDP cells into nude mice. **b**, **c**. Effects of Pt and Pt-MWNTS on tumor volume and tumor weight in nude mice. **d** Effects of Pt and Pt-MWNTS on nude mice body weight. (n = 6, *P < 0.05*,* **P < 0.01, ***P < 0.001 vs Control). **e** Immunohistochemical images of tumor sections in the Pt and Pt-MWNTS groups were shown. CD31 was used to indicate the blood vessels in the tumor, and TUNEL was used to show the cell apoptosis in the tumor

We then verify the ability of Pt-MWNTS on NSCLC tumorigenesis.

in nude mice model (Fig. 6a). A549/DDP cells were subcutaneously injected into 20 nude mice and were randomly divided into 4 groups. Cisplatin and Pt-MWNTS were delivered to nude mice by intravenous injection the next day. After about 3 weeks, mice receiving cisplatin (Pt) and Pt-MWNTS exhibited smaller tumor volume and tumor weight than control group and MWNTS group (Fig. 6b, c). Moreover, the inhibition effects of Pt-MWNTS was stronger than cisplatin. Both of them have no significant effect on body weight (Fig. 6d). Immunohistochemical images of tumor sections indicated less blood vessels and stronger apoptosis in Pt-MWNTS treated group than free cisplatin (Pt) (Fig. 6e). Therefore, our data suggested that Pt-MWNTS inhibited tumorigenesis in vivo, and the efficiency was better than free cisplatin.

## Discussion

Lung cancer is a leading cause of cancer-related deaths over the world. Multiple kinds of Chemotherapeutics such as cis-platinum are currently available for lung cancer treatment, while eventually none of them can escape from drug-resistance of tumor cells, which can limit their anti-tumor efficacy. Thus, providing novel strategies for overcoming platinum-resistance is of great importance. In this study, we used MWNTS as a carrier to deliver cisplatin. We found that application of Pt- MWNTS can significantly promote the apoptosis of human cisplatin-resistant lung adenocarcinoma cell line A549/DDP and inhibit the expression of EMT marker proteins and downstream transcription factors, and thus suppress the migration and invasion ability of cancer cells in vitro. In vivo study further confirmed that Pt- MWNTS can inhibited the tumorigenesis of A549/DDP. Therefore, our data suggested that Pt-MWNTS could reverse the drug resistance of cisplatin in lung cancer.

Platinum compounds were widely used in chemotherapy of numerous human cancers and drug resistance has always been one of the therapeutic challenges. The underlying mechanism of drug resistance were complicated. Copper efflux transporters ATP7A and ATP7B were reported playing an important role in platinum drug resistance [11]. Besides, EZH2 protein was found to be over-expressed in drug-resistant cancer cells and a nanoparticle system containing siEZH2 can reverse cisplatin resistance [34]. It is said that Cyclooxygenase-2 (COX-2) promotes ovarian cancer cell cisplatin resistance by regulating EMT progress [35]. In this study, we found that Pt- MWNTS can reverse cisplatin resistance by regulating EMT in A549/DDP. Multiwalled carbon nanotubes have been identified as an efficient drug carrier [36], and was first explored having potential to enhance treatment of cancer cells by coupling with laser irradiation [37]. Study has shown that MWNTs exhibited no toxicity when it used alone, but dramatically decreased cell viability when combined with laser irradiation [38]. In our study, we use MWNTs as a carrier of cisplatin to reverse the drug assistance of NSCLC. We found that, combination of cisplatin and MWNT significantly promoted the accumulation of cisplatin in cells.

Platinum compounds are widely used chemotherapeutic agents, but in our study, we only study Pt-MWNTS effects on NSCLC, whether it can reverse drug resistance of other cancer like ovarian cancer needs further study. It is said that use of dose-intense or high-dose chemotherapy was correlated with better progression free survival [39], thus finding a proper dose of Pt-MWNTS for clinical study is of great importance in the future.

## Conclusions

In summary, we report for the first time that MWNT loaded Platinum eventually achieve reverse drug resistance by inhibiting EMT in NSCLC. Pt- MWNT reverses the drug resistance in the A549/DDP cell line, underlying its possibility of treating NSCLC with cisplatin resistance.

## Supplementary Information

**Additional File 1: Fig. S1** The unprocessed western blot images of figure 3C. A-C were the stripping and reprobing of the same membrane using different antibodies. D-I were the stripping and reprobing of a second membrane using different antibodies.

**Additional file1: Fig. S1** The unprocessed western blot images of figure3C. A-C were the stripping and reprobingof the same membrane using different antibodies. D-Iwere the stripping and reprobingofa second membrane using different antibodies (JPG 151 KB)

## Data Availability

The datasets used and/or analyzed during the current study are available from the corresponding author on reasonable request.

## Abbreviations

NSCLC: Non-small-cell lung cancer
EMT: Epithelial-mesenchymal transformation
MT: Metallothionein
CNTs: Carbon nanotubes
NMs: Nanomaterials
MWCNTs: Multiwall carbon nanotubes
COX-2: Cyclooxygenase-2

## References

[1] Xu F, Chen JX, Yang XB, Hong XB, Li ZX, Lin L, Chen YS (2020). Analysis of lung adenocarcinoma subtypes based on immune signatures identifies clinical implications for cancer therapy. Mol Ther-Oncolytics.

[2] Wang Y, Guo S, Li D, Tang Y, Li L, Su L, Liu X (2020). YIPF2 promotes chemotherapeutic agent-mediated apoptosis via enhancing TNFRSF10B recycling to plasma membrane in non-small cell lung cancer cells. Cell Death Dis.

[3] Chen J, Liu A, Lin Z, Wang B, Chai X, Chen S, Lu W, Zheng M, Cao T, Zhong M (2020). Downregulation of the circadian rhythm regulator HLF promotes multiple-organ distant metastases in non-small cell lung cancer through PPAR/NF-κb signaling. Cancer Lett.

[4] Arbour KC, Riely GJ (2019). Systemic therapy for locally advanced and metastatic non-small cell lung cancer: a review. JAMA.

[5] Xing DF, Xu CD, Liao XY, Xing TY, Cheng SP, Hu MG, Wang JX (2019). Spatial association between outdoor air pollution and lung cancer incidence in China. BMC Public Health.

[6] Sullivan DR, Eden KB, Dieckmann NF, Golden SE, Vranas KC, Nugent SM, Slatore CG (2019). Understanding patients' values and preferences regarding early stage lung cancer treatment decision making. Lung Cancer.

[7] Gong WJ, Ma LY, Hu L, Lv YN, Huang H, Xu JQ, Huang DD, Liu RJ, Han Y, Zhang Y (2019). STAT3 rs4796793 contributes to lung cancer risk and clinical outcomes of platinum-based chemotherapy. Int J Clin Oncol.

[8] Zhang X, Qin T, Zhu Z, Hong F, Xu Y, Zhang X, Xu X, Ma A (2020). Ivermectin augments the in vitro and in vivo efficacy of cisplatin in epithelial ovarian cancer by suppressing Akt/mTOR signaling. Am J Med Sci.

[9] Dong XL, Gong Y, Chen ZZ, Wang YJ (2014). Delisheng Injection (), a Chinese medicinal compound, enhanced the effect of cis-platinum on lung carcinoma cell line PGCL3. Chin J Integr Med.

[10] Schmitt NC, Page BR (2018). Chemoradiation-induced hearing loss remains a major concern for head and neck cancer patients. Int J Audiol.

[11] Li YQ, Yin JY, Liu ZQ, Li XP (2018). Copper efflux transporters ATP7A and ATP7B: Novel biomarkers for platinum drug resistance and targets for therapy. IUBMB Life.

[12] Gao J, Wang W (2019). Knockdown of galectin-1 facilitated cisplatin sensitivity by inhibiting autophagy in neuroblastoma cells. Chem-Biol Interact.

[13] Ishida S, Lee J, Thiele DJ, Herskowitz I (2002). Uptake of the anticancer drug cisplatin mediated by the copper transporter Ctr1 in yeast and mammals. P Natl Acad Sci USA.

[14] Kalayda GV, Wagner CH, Buss I, Reedijk J, Jaehde U (2008). Altered localisation of the copper efflux transporters ATP7A and ATP7B associated with cisplatin resistance in human ovarian carcinoma cells. BMC Cancer.

[15] Ushijima R, Takayama K, Izumi M, Harada T, Horiuchi Y, Uchino J, Hara N, Nakanishi Y (2007). Immunohistochemical expression of MRP2 and clinical resistance to platinum-based chemotherapy in small cell lung cancer. Anticancer Res.

[16] Zhu Z, Du S, Du Y, Ren J, Ying G, Yan Z (2018). Glutathione reductase mediates drug resistance in glioblastoma cells by regulating redox homeostasis. J Neurochem.

[17] Lee JH, Chae JW, Kim JK, Kim HJ, Chung JY, Kim YH (2015). Inhibition of cisplatin-resistance by RNA interference targeting metallothionein using reducible oligo-peptoplex. J Control Release.

[18] Wynne P, Newton C, Ledermann JA, Olaitan A, Mould TA, Hartley JA (2007). Enhanced repair of DNA interstrand crosslinking in ovarian cancer cells from patients following treatment with platinum-based chemotherapy. Brit J Cancer.

[19] Sawant A, Kothandapani A, Zhitkovich A, Sobol RW, Patrick SM (2015). Role of mismatch repair proteins in the processing of cisplatin interstrand cross-links. DNA Repair.

[20] Bao L, Wu J, Dodson M, Rojo de la Vega EM, Ning Y, Zhang Z, Yao M, Zhang DD, Xu C, Yi X (2017). ABCF2, an Nrf2 target gene, contributes to cisplatin resistance in ovarian cancer cells. Mol Carcinogen..

[21] Zhou F, Yang X, Zhao H, Liu Y, Feng Y, An R, Lv X, Li J, Chen B (2018). Down-regulation of OGT promotes cisplatin resistance by inducing autophagy in ovarian cancer. Theranostics.

[22] Xin L, Zhou Q, Yuan YW, Zhou LQ, Liu L, Li SH, Liu C (2019). METase/lncRNA HULC/FoxM1 reduced cisplatin resistance in gastric cancer by suppressing autophagy. J Cancer Res Clin Oncol.

[23] Zhang X, Qi Z, Yin H, Yang G (2019). Interaction between p53 and Ras signaling controls cisplatin resistance via HDAC4- and HIF-1α-mediated regulation of apoptosis and autophagy. Theranostics.

[24] Marcucci F, Stassi G, De Maria R (2016). Epithelial-mesenchymal transition: a new target in anticancer drug discovery. Nat Rev Drug Discov.

[25] Ren P, Zhang H, Chang L, Hong XD, Xing L (2020). LncRNA NR2F1-AS1 promotes proliferation and metastasis of ESCC cells via regulating EMT. Eur Rev Med Pharmaco..

[26] Han ML, Zhao YF, Tan CH, Xiong YJ, Wang WJ, Wu F, Fei Y, Wang L, Liang ZQ (2016). Cathepsin L upregulation-induced EMT phenotype is associated with the acquisition of cisplatin or paclitaxel resistance in A549 cells. Acta Pharmacol Sin.

[27] Qiu E, Gao Y, Zhang B, Xia T, Zhang Z, Shang G (2020). Upregulation of cell division cycle 20 in cisplatin resistance-induced epithelial-mesenchymal transition in osteosarcoma cells. Am J Transl Res.

[28] Yang J, Xing H, Lu D, Wang J, Li B, Tang J, Gu F, Hong L (2019). Role of Jagged1/STAT3 signalling in platinum-resistant ovarian cancer. J Cell Mol Med.

[29] Miyazaki H, Takahashi RU, Prieto-Vila M, Kawamura Y, Kondo S, Shirota T, Ochiya T (2018). CD44 exerts a functional role during EMT induction in cisplatin-resistant head and neck cancer cells. Oncotarget..

[30] Kumar MK, Ramaprabhu S (2006). J Mater Chem B.

[31] Cao Y, Luo Y (2019). Pharmacological and toxicological aspects of carbon nanotubes (CNTs) to vascular system: a review. Toxicol Appl Pharm.

[32] Wang X, Li B, Jing H, Dong X, Leng X (2020). MWCNT-mediated combinatorial photothermal ablation and chemo-immunotherapy strategy for the treatment of melanoma. J Mater Chem B.

[33] Dong X, Sun Z, Wang X, Zhu D, Liu L, Leng X (2017). Simultaneous monitoring of the drug release and antitumor effect of a novel drug delivery system-MWCNTs/DOX/TC. Drug Deliv.

[34] Yu C, Ding B, Zhang X, Deng X, Deng K, Cheng Z, Xing B, Jin D, Ma P, Lin J (2018). Targeted iron nanoparticles with platinum-(IV) prodrugs and anti-EZH2 siRNA show great synergy in combating drug resistance in vitro and in vivo. Biomaterials.

[35] Deng L, Feng DQ, Ling B (2020). Cyclooxygenase-2 promotes ovarian cancer cell migration and cisplatin resistance via regulating epithelial mesenchymal transition. J Zhejiang Univ-Sc B.

[36] Anbarasan B, Babu SV, Elango K, Shriya B, Ramaprabhu S (2015). pH responsive release of doxorubicin to the cancer cells by functionalized multi-walled carbon nanotubes. J Nanosci Nanotechno.

[37] Fisher JW, Sarkar S, Buchanan CF, Szot CS, Whitney J, Hatcher HC, Torti SV, Rylander CG, Rylander MN (2010). Photothermal response of human and murine cancer cells to multiwalled carbon nanotubes after laser irradiation. Cancer Res.

[38] Lin Z, Liu Y, Ma X, Hu S, Zhang J, Wu Q, Ye W, Zhu S, Yang D, Qu D (2015). Photothermal ablation of bone metastasis of breast cancer using PEGylated multi-walled carbon nanotubes. Sci Rep-UK.

[39] Alifrangis C, Lucas O, Benafif S, Ansell W, Greenwood M, Smith S, Wilson P, Thomas B, Rudman S, Mazhar D (2020). Management of late relapses after chemotherapy in testicular cancer: optimal outcomes with dose-intense salvage chemotherapy and surgery. Eur Urol Focus.

