# Supplementary figures and images for: Cisplatin loaded multiwalled carbon nanotubes reverse drug resistance in NSCLC by inhibiting EMT
Source: Cancer Cell Int. 2021 Jan 25;21:74. doi: 10.1186/s12935-021-01771-9 (PMC7836500; doi:10.1186/s12935-021-01771-9)

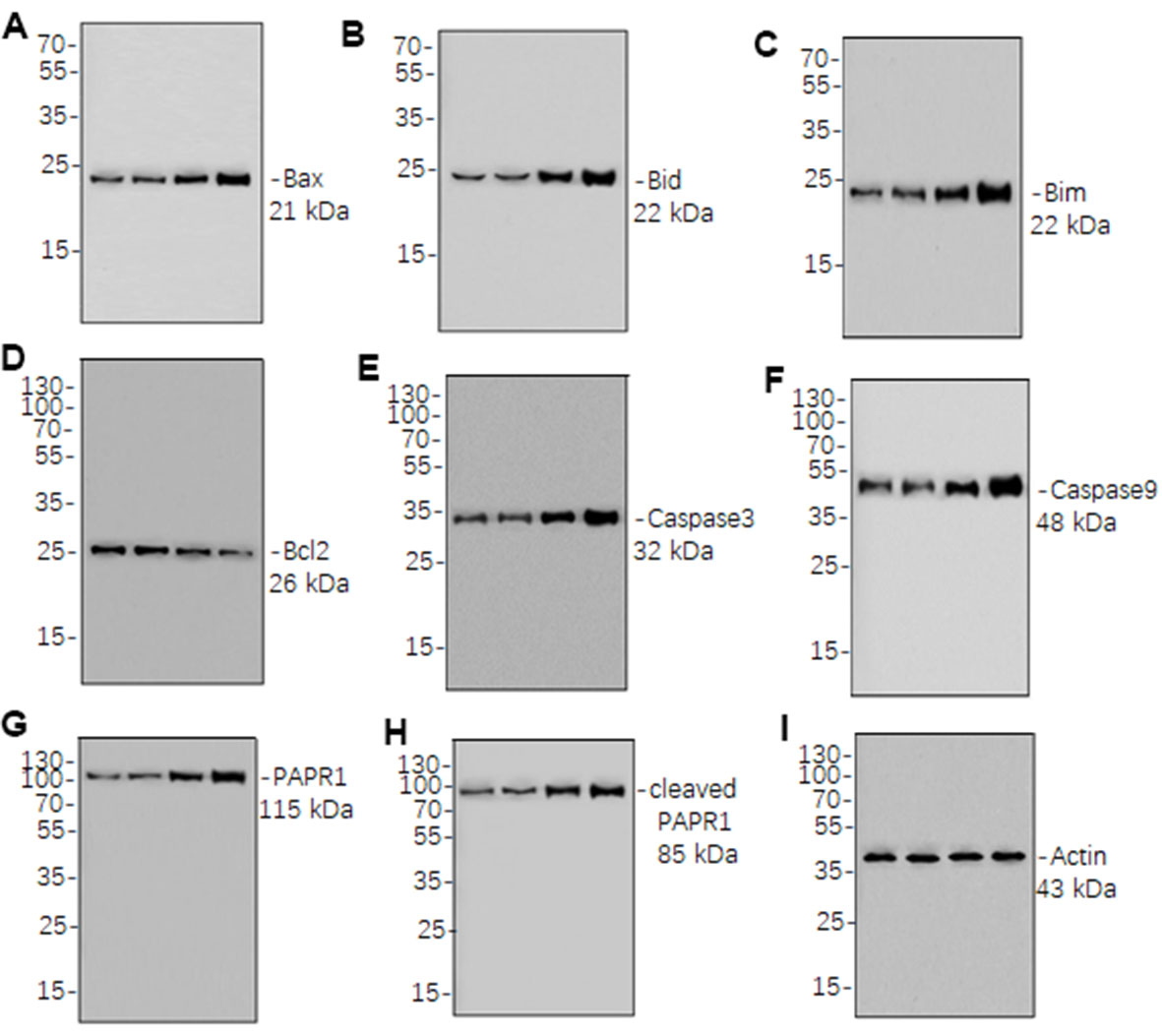

Supplement: Supplementary file 1 — Additional file1: Fig. S1 The unprocessed western blot images of figure3C. A-C were the stripping and reprobingof the same membrane using different antibodies. D-Iwere the stripping and reprobingofa second membrane using different antibodies (JPG 151 KB) [file 12935_2021_1771_MOESM1_ESM.jpg]
